# Molecular distribution in intradermal injection for transfer and delivery of therapeutics

**DOI:** 10.3389/fddev.2023.1095181

**Published:** 2023-01-13

**Authors:** Emran O. Lallow, Kishankumar J. Busha, Sarah H. Park, Maria Atzampou, Nandita C. Jhumur, Yasir Demiryurek, Christine C. Roberts, Jerry W. Shan, Jeffrey D. Zahn, David I. Shreiber, Young K. Park, Jonathan P. Singer, Joel N. Maslow, Hao Lin

**Affiliations:** ^1^ Department of Mechanical and Aerospace Engineering, Rutgers, The State University of New Jersey, Piscataway, NJ, United States; ^2^ Department of Biomedical Engineering, Rutgers, The State University of New Jersey, Piscataway, NJ, United States; ^3^ Department of Materials Science and Engineering, Rutgers, The State University of New Jersey, Piscataway, NJ, United States; ^4^ Department of Mechanical Engineering, Temple University, Philadelphia, PA, United States; ^5^ GeneOne Life Science Inc., Seoul, South Korea

**Keywords:** DNA-based vaccines, intradermal injection, molecular delivery, molecular distribution, transfection

## Abstract

Intradermal (ID) injection is a technique widely used in laboratorial and clinical applications. The boundary of the dome-like bleb formed during injection is assumed to represent the lateral extent of the injected material. This work systematically characterizes cargo molecule distribution (puddle) as a function of injection volume and molecular/particle size in rat skin post ID injection. In general, results indicate that the puddle forms a subdomain laterally contained within the bleb, with an area inversely correlating to the molecular size of the injected material. For 50 μL and 100 µL injections, the average area of the bleb was 40.97 ± 6.30 mm^2^ and 55.64 ± 8.20 mm^2^, respectively, regardless of the molecular/particle size. On the other hand, the area of the puddle was dependent on the molecular size and ranged between 45.38 ± 8.29 mm^2^ and 6.14 ± 4.50 mm^2^ for 50 µL injections, and 66.64 ± 11.22 mm^2^ and 11.50 ± 9.67 mm^2^ for 100 µL injections. The lateral distribution appears to have no time-dependency up to 10 min post injection. The trend in the depth of cargo penetration is also similar, with smaller particles extending deeper into the dermis and subcutaneous fat layers. Because the area of puddle can be significantly less than that of the bleb, establishing base characterization is essential to understand cellular interactions with the injected biological substances.

## Introduction

**FIGURE 1:**
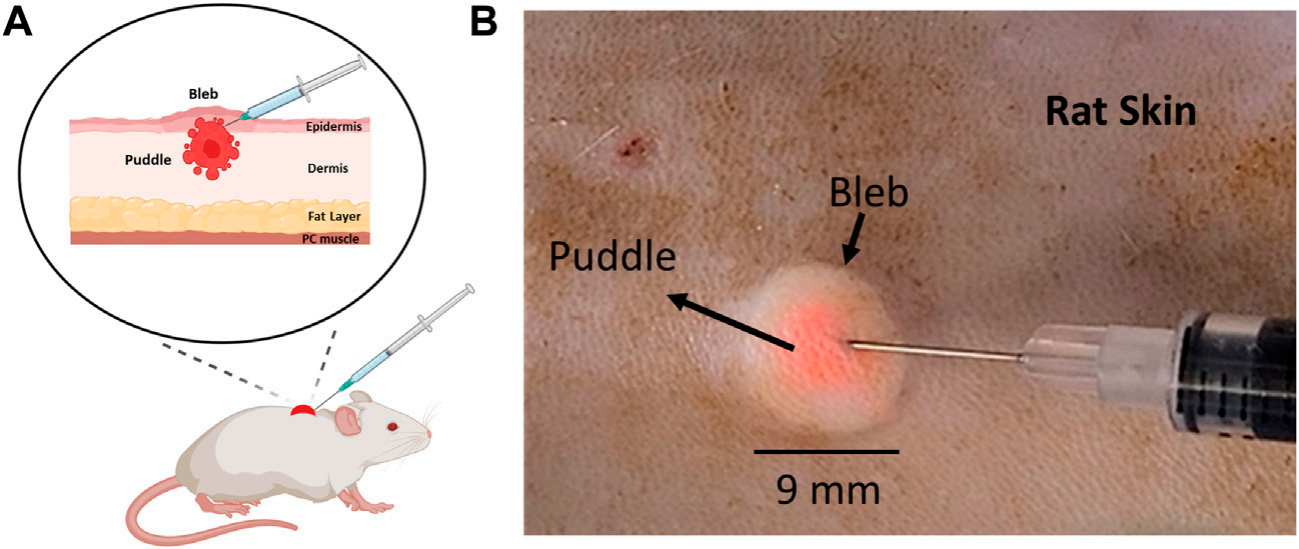
Location of the injected cargo (puddle) in relation to the injection bleb post ID injection. **(A)** A schematic diagram of a rat skin model indicating the location of the puddle relative to the bleb. **(B)** 100 µL ID injection of 3 µm fluorescent particles in rat skin.

The intradermal (ID) Mantoux injection technique, first introduced in the early 20th century to deliver purified protein derivative (PPD) to diagnose *tuberculosis*, has found its way into wide applications in administering therapeutics and bioactive materials (Laurent et al., 2007; Lambert and Laurent, 2008; Tanizaki et al., 2015). This technique is performed by inserting the tip of a 33 to 26-gauge needle parallel to the skin to target the dermal layer. The drug is then injected into the skin to form a light-colored, dome-like marking, herein referred to as the “bleb” (Laurent et al., 2007; Lambert and Laurent, 2008; Zehrung et al., 2013; Chuaychoo et al., 2020) (Figure 1). Presently, the ID route is employed to administer common medications including heparin, insulin, growth hormones, interferons, and antibodies, with advantages comparable to the intramuscular (IM) route (Usach et al., 2019). It is also used to deliver allergen specific immunotherapy to treat several IgE-mediated food allergies and is being implemented in cancer therapies for melanoma treatments, where Interleukin-2 (IL-2) is injected onto the cutaneous lesion (Byers et al., 2014; Senti and Kündig, 2016; Sloot et al., 2016). Another significant utility of ID injections is to administer vaccines, with multiple clinical studies demonstrating several advantages for the ID route, including strong immune responses, optimum dosing effects, patient tolerability, and cost-effectiveness (Lambert and Laurent, 2008; Zehrung et al., 2013; Tanizaki et al., 2015; Meunier et al., 2016; Roozen et al., 2022). To date, many vaccines implementing ID injection have been approved for clinical testing, including vaccines for the seasonal influenza, yellow fever, inactivated poliovirus (IPV), rabies, hepatitis B, and COVID-19 (Laurent et al., 2007; Lambert and Laurent, 2008; Zehrung et al., 2013; Schaumburg et al., 2019; Momin et al., 2021). A key consideration for intradermally administered biologic substances that act locally is that effectiveness is critically dependent on the molecular dispersion within the volume of cells surrounding the injection site.

One important question that has been poorly studied with regard to the application of ID injection is the actual lateral distribution of the “cargo” (therapeutics and bioactive materials) post injection. In the absence of further evidence, the obvious choice is to regard the bleb as the lateral extent of cargo, as the cargo is typically dissolved in the liquid that distends to cause the bleb. While this may be reasonable for smaller molecular cargos, larger molecules may have limited mobility and dispersion range within the tissue post injection. One example is the treatment of melanoma and other skin lesions for which bioactive molecules, such as monoclonal antibodies or cytokines are injected directly into the skin (Byers et al., 2014; Sloot et al., 2016). This issue becomes particularly important for cutaneous nucleic acid delivery where the cargo molecular size is typically in the million-Dalton range, with protein expression proportional to the number of transfected cells. Moreover, because a secondary mechanism is required to drive or enhance cellular uptake, it is imperative to clearly co-localize the applied transfection device with the injected DNA for maximal effect. For example, in a recent new technique developed by our group to administer DNA-based vaccines, we have demonstrated that applying a moderate negative pressure atop of the injection bleb caused by ID administration produces a strong humoral and cellular immune responses (Lallow et al., 2021; Jeong et al., 2022). The cutaneous application of negative pressure enhances cellular uptake, allowing DNA vectors to permeate across the cell membrane, and ultimately localize within the cell nucleus, so as to express and export encoded proteins. A similar, yet more commonly used approach is electroporation, which utilizes electric pulses to permeabilize the membrane to initiate endocytosis (Ake et al., 2017; Thottacherry et al., 2018; Gary and Weiner, 2020; Dong and Chang, 2021; Lallow et al., 2021; Jeong et al., 2022). Common to both approaches is the so-called “colocalization” principle: the secondary mechanism, be it negative pressure or electric field application, needs to be activated where the “cargo” (DNA plasmids) is located so that they may traverse the membrane barrier into neighboring cells. The number of cells transfected within the “colocalization region” should directly correlate with the number of cells in direct contact with the DNA plasmids. Intuitively, this points to the injection bleb (Figure 1) as a visual target onto which the secondary mechanism is applied (Denet et al., 2004; Van Den Berg et al., 2009; Kis et al., 2012; Amante et al., 2015; Lallow et al., 2021; Lawal et al., 2022). The main motivation of the current work is therefore to characterize cargo distribution extent (termed “puddle”, Figure 1A) relative to the bleb, with the hypothesis that larger molecular size leads to appreciable difference between bleb and puddle extents.

We perform a systematic examination of cutaneous cargo distributions post ID injection by utilizing four distinctly sized molecules with different injection volumes. This study will provide important information so as to best establish “colocalization regions” for the purposes of optimizing post injection cutaneous transfection methods or understanding the distribution of bioactive materials that require regional localization or cellular uptake for function.

## Materials and methods

### Animals

Adult male Sprague Dawley rats (NTac-SD; murine pathogen free), purchased from Taconic Biosciences, Inc. (Germantown, NY), were housed under controlled conditions (12-h:12-h light-dark cycle, room temperature) and in accordance with the guidelines established by the Rutgers University Institutional Animal Care and Use Committee under protocol IACUC-201800077. Rats were sacrificed prior to experiment and shaved with a hair clipper (WAHL, Sterling, Illinois). Hair removal cream (Nair hair removal lotion, Nair) was applied to the skin for 3–5 min and cleaned thoroughly with 70% ethanol. All experiments were performed within an hour of animal sacrifice to ensure both the integrity and viability of the skin (Oesch et al., 2014; Kocsis et al., 2022).

### Injection materials

3–5 kDa FITC-dextran (∼1 nm) (Armstrong et al., 2004) (Fluorescein Isothiocyanate-dextran, Millipore Sigma, Burlington, MA) was diluted to a concentration of 200 µM in 1× PBS. Spherical fluorescent particles with 20 nm and 3.0 µm (3,000 nm) diameter (Thermo Scientific™ Dyed Red Aqueous Fluorescent Particles, Fisher Scientific, Waltham, MA) were used at concentrations of 1.05 and .525 g/cm^3^, respectively, in deionized water. Particles were briefly vortexed prior to use to ensure homogenous dispersion. pEGFP-N1 DNA plasmid (∼100 nm, see details in *Results*) was provided by GeneOne Life Science (Seoul, South Korea) and was diluted to a concentration of 1 mg/mL in 1× PBS solution. Plasmid DNA was labeled with Cy5 dye (Label IT Nucleic Acid Labeling Kit Cy5, Mirus Bio, Madison, WI) at a 1:1 ratio, incubated for 1 h at 37°C per manufacturer’s instruction, and diluted to a final concentration of .01 mg/mL using nuclease-free water (Invitrogen™ Nuclease-Free Water, Fisher Scientific, Waltham, MA).

### DNA size measurement

The size of DNA was determined by dynamic light scattering measurements (DLS). 3-4 wells of a 96-well plate (Elplasia 96-well Plate, Corning, Corning, NY) were loaded with 70 µL of Cy5-labeled DNA at a concentration of .01 mg/mL or 70 µL of unlabeled DNA (pEGFP-N1) at a concentration of .25 mg/mL. Samples were placed inside a plate reader (DynaPro Plate Reader III, Wyatt Technology, Santa Barbara, CA), and 10 acquisitions were obtained for each well. GraphPad Prism software (9.0.0, GraphPad Software, San Diego, CA) was used to remove outliers using the ROUT method (Q = 1%).

### Experimental protocols

The Mantoux ID injection technique was performed to inject either 50 µL or 100 µL of each material into the epidermal-dermal junction and the upper dermis using a 28-gauge insulin syringe (Micro-Fine™ IV Insulin Syringes, BD Franklin Lakes, NJ). Skin was excised shortly after injection or after 10 min to assess the effect of collection time on molecular dispersion.

For Figures 3C–J, 1.5 × 1.5 cm^2^ samples of excised skin were embedded in optimal cutting temperature compound (OCT) and cryosectioned vertically to a thickness of 60 µm (CM3050S, Leica, Buffalo Grove, IL). Every fifth section was collected.

### Imaging and characterization

Bright field images of the injection bleb were collected after injection using a smartphone camera (Galaxy S20, Samsung, Seoul, South Korea), and Fiji software (Fiji: an open-source platform for biological-image analysis, NIH, Bethesda, Maryland) was used to obtain area measurements. Three replicate measurements for the area of the bleb were performed and averaged. Fluorescent images of the puddle and the sections were collected with a ×4 objective on the FITC, Cy5, and TRITC channels for the dextran, labeled DNA, and fluorescent particles, respectively (Eclipse TE2000-S, Nikon, Tokyo, Japan; and Plan Fluor, Nikon, Tokyo, Japan). A MATLAB algorithm was developed to correct for autofluorescence signal. The radial intensity profile for each image was determined by averaging the radial intensity of 12 equidistant radial lines spanning the area of the puddle. By inspecting the logarithmic transformation of the radial intensity profiles, an 80% high-pass filter was applied to consistently correct for autofluorescence across all cases. The area of the puddle was then calculated by converting the pixel size to area units (R2021b, MathWorks, Natick, MA). Supplementary Figure S1 shows exemplary results from the algorithm.

All graphs were generated using GraphPad Prism software, except for graphs in Figure 5, which were generated using MATLAB.

## Statistics

All data in this study is represented by mean ± SD. An ordinary one-way ANOVA followed by a Tukey’s multiple comparisons test or a standard Student’s t-test were performed on the data using GraphPad Prism software. Data significance was determined with a value of *p* ≤ .05.

## Results

Figure 1A shows a schematic post ID injection that represents an exemplary scenario where the cargo puddle is smaller than the bleb. This is experimentally demonstrated in Figure 1B, where a 100 µL solution of 3 µm-diameter fluorescent particles were injected. The bright field image shows that the particles (pink-colored) are concentrated at the center of the bleb, occupying a significantly smaller mean area (∼11.5 mm^2^) when compared with the latter (∼55 mm^2^). In addition, the area of the injected cargo was centered around the needle tip as the bleb formed during injection. Movie M1 demonstrates this process.

To assess the relative lateral dispersion in tissue of injected material relative to molecular size, we assessed two commercially purchased size specified fluorescent particles, a small biomolecule (FITC-dextran), and Cy5-labeled DNA. The diameter the pEFGP-N1 and Cy5-labeled pEGFP-N1 were determined as 149.9 ± 28.47 nm (34 measurements) and 99.83 ± 14.45 nm (27 measurements), respectively, with the size of unlabeled pEGFP-N1 conforming to work published by others for a plasmid of 4,700 bp (Latulippe and Zydney, 2010; Li et al., 2012). DLS measurements for DNA plasmids are presented in Supplementary Figure S2. The four materials used in this study spanned a range from 1 nm to 3,000 nm in diameter and are summarized in Table 1.

**TABLE 1:** Summary of molecular cargo information. The diameter of Cy5-labeled DNA is measured to be 99.83 ± 14.45 nm (n = 27) per our experiment, whereas the sizes of FITC-dextran and fluorescent particles are adopted from prior work (Latulippe and Zydney, 2010) and provided by manufacturer, respectively.

Material	MW	Size (diameter)	Concentration	Diluent
FITC-dextran	∼3–5 kDa	∼1 nm	200 µM	1× PBS
Dyed Red Aqueous Fluorescent Particles (20 nm)	—	20 nm	1.05 g/cm^3^	DI water
Cy5-labeled DNA (pEGFP-N1)	∼10^3^ kDa	∼100 nm	.01 mg/mL	Nuclease-free water
Dyed Red Aqueous Fluorescent Particles (3.0 µm)	—	3,000 nm	.525 g/cm^3^	DI water

**FIGURE 2:**
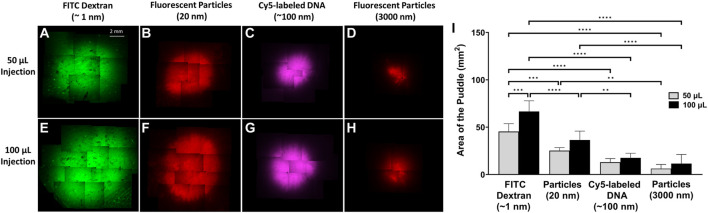
Puddle area dependence on molecular/particle size and injection volume. ×4 objective top-view fluorescent images of the puddle post ID injections. **(A–D)** 50 µL solution. **(E–H)** 100 µL solution. **(I)** Areas of the puddle resulting from 50 to 100 µL injections for the different molecules/particles. *n =* 6 each. Data represents mean ± SD. Statistics are presented as **p ≤ .01, ***p ≤ .001, and ****p ≤ .0001 by ordinary one-way ANOVA followed by a Tukey’s multiple comparisons test.

To illustrate the dependence of the puddle area on the size and volume of the injected material, each of the four different molecules and/or particles were injected at either 50 µL or 100 µL volumes, and puddle areas were quantified immediately after injection. The top row of Figures 2A–D shows that for a 50 µL injection, the area of the puddle decreases as the size of the injected material increases. A similar trend follows when injecting 100 μL, which is shown in the bottom row of Figures 2E–H. Figure 2I shows area quantification of the puddle area, indicating that a higher injection volume on average leads to a larger puddle area. However, the only statistical difference in the puddle size between the 50 μL and 100 µL injections occurs for ∼1 nm-diameter FITC-dextran. Across materials, the areas of the puddle for FITC-dextran injections (both 50 μL and 100 µL) were significantly different when compared with Cy5-labeled DNA and both 20 nm and 3 µm fluorescent particles’ puddles. The 50 µL injection puddle size for 20 nm fluorescent particles was statistically different compared to the 3 µm particles, and the 100 µL injection puddle for 20 nm fluorescent particles was statistically different compared to the same injection for Cy5-labeled DNA and 3 µm particles.

**FIGURE 3:**
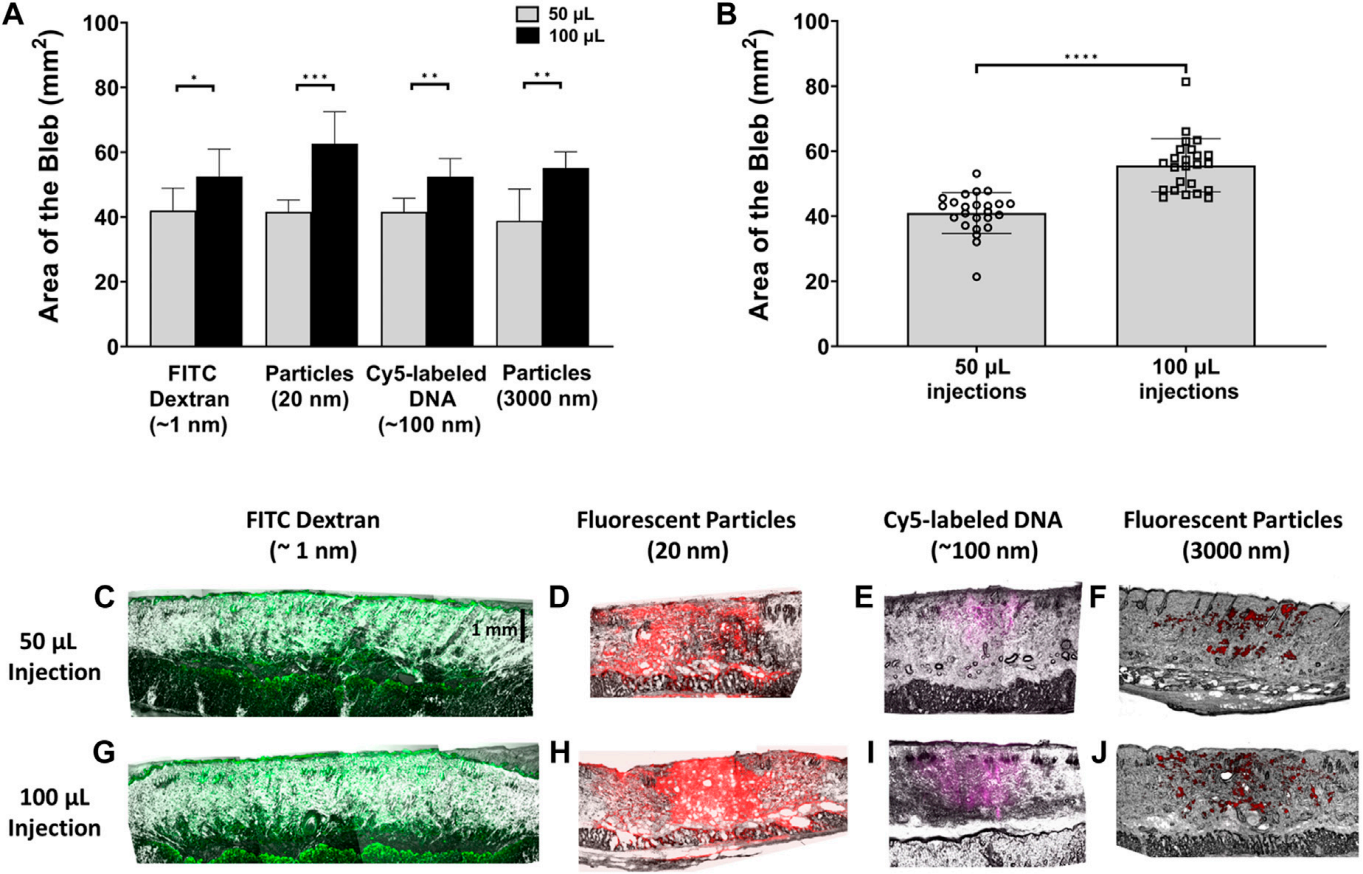
Quantification of bleb area and cargo depth with respect to injection volume. **(A)** Bleb area comparison for 50 μL and 100 µL injections. *n =* 6 each. **(B)** Area of injection bleb for 50 μL and 100 µL volumes, combining the areas of all injected materials for each respective volume. *n =* 24 each. Data represents mean ± SD. *p ≤ .05, **p ≤ .01, ***p ≤ .001, and ****p ≤ .0001 by Student’s t-test. Panels **(C–J)** represent photomicrograph of fluorescent signal in vertically sectioned skin samples post ID injection. Images were collected with a ×4 objective for 60-µm thick rat skin sections post 50 µL **(C–F)** and 100 µL **(G–J)** injections of the different cargo molecules/particles.

For all materials with the same injection volume, there were no significant differences among the area of all the injection blebs compared to each other regardless of the injected material’s size (Figure 3A). However, the area of the bleb is significantly larger for 100 µL injections compared to 50 µL injections for all materials. Figure 3B shows combined bleb areas (n = 24) for all 50 μL and 100 µL injection. The bleb area of the 100 µL injection was significantly different than the bleb area of the 50 µL injection. To obtain a visualization of the injected cargo within the bleb in the vertical direction, immediately after injection, the depth of the puddle was investigated for both 50 and 100 µL injections. In Figures 3C, G, the depth of the puddle of the FITC-dextran is observed within the fat layer of the skin and thus appears to reach a depth >2 mm. This was also the case for 20 nm fluorescent particles in Figures 3D, H. Puddle penetration depths were visibly shallower for Cy5-labeled DNA (Figures 3E, I) and 3 µm fluorescent particles (Figures 3F, J), where for the latter the distribution appears to be more sporadic within the skin. The vertical distribution of the puddle appears to depend only weakly on the injection volume. In fact, using that the bleb area increases 36% (Figure 3B) with injection volume doubling, we can estimate a bleb height increase of 47%. This significant increase in bleb height only caused a minor effect on cargo depth, more notably for larger-size molecules. On the other hand, puddle penetration depth does depend much more strongly on the size of the injected material in a manner similar to observations from a top view such as in Figure 2. That is, puddle size is inversely correlated with particle size.

**FIGURE 4:**
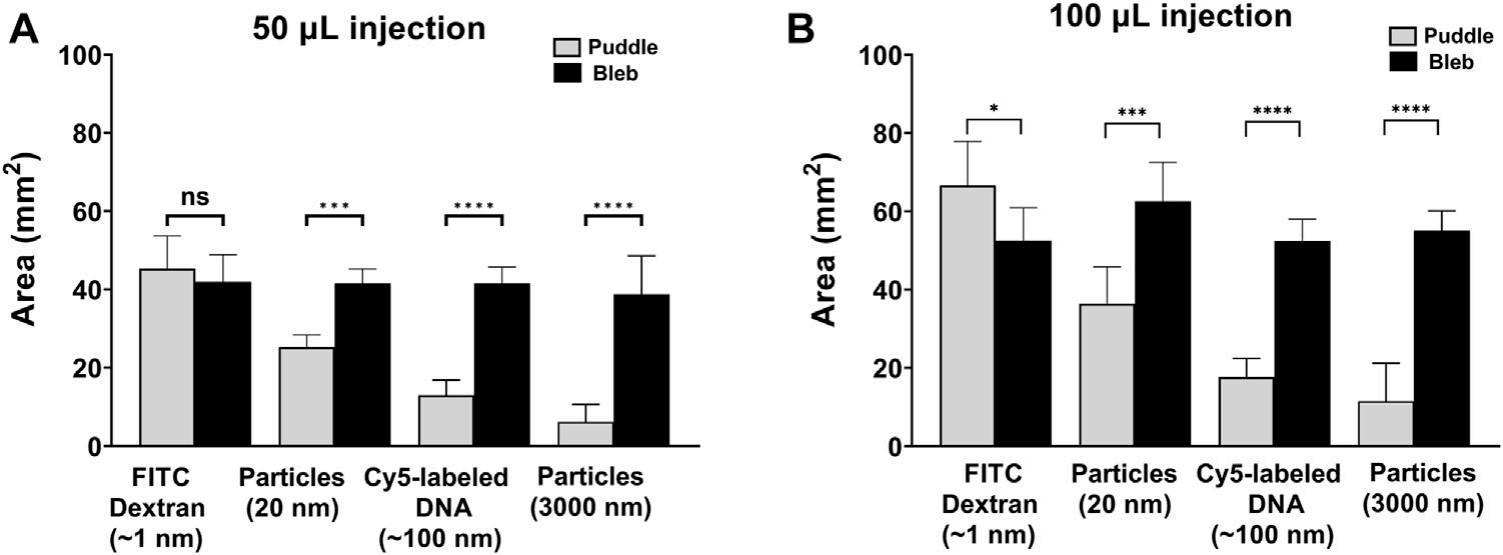
Area quantification of the puddle and the bleb. Areas of the puddle and bleb using **(A)** 50 µL and **(B)** 100 µL injections. *n =* 6 each. Data represents mean ± SD. *p ≤ .05, **p ≤ .01, ***p ≤ .001, and ****p ≤ .0001 by Student’s t-test.

To further demonstrate the relative size of the puddle in comparison to the bleb, Figure 4 shows quantitative analyses for the bleb and the puddle areas for 50 µL (Figure 4A) and 100 µL injections (Figure 4B). In Figure 4A, the area of the FITC-dextran puddle matches the area of the bleb. However, as the molecular/particle size of the injected cargo increases, the area of the puddle becomes significantly smaller than that of the bleb. For ID injections of 100 µL (Figure 4B), it is peculiar to note that the puddle area for FITC-dextran is larger than the bleb, which indicates that the injected solution exceeds the boundary of the bleb dispensing through a larger area of the skin. 100 µL injections for Cy5-labeled DNA and both fluorescent particles follow the same trend as the 50 µL injections but with a larger mean area for both the bleb and the puddle.

**FIGURE 5:**
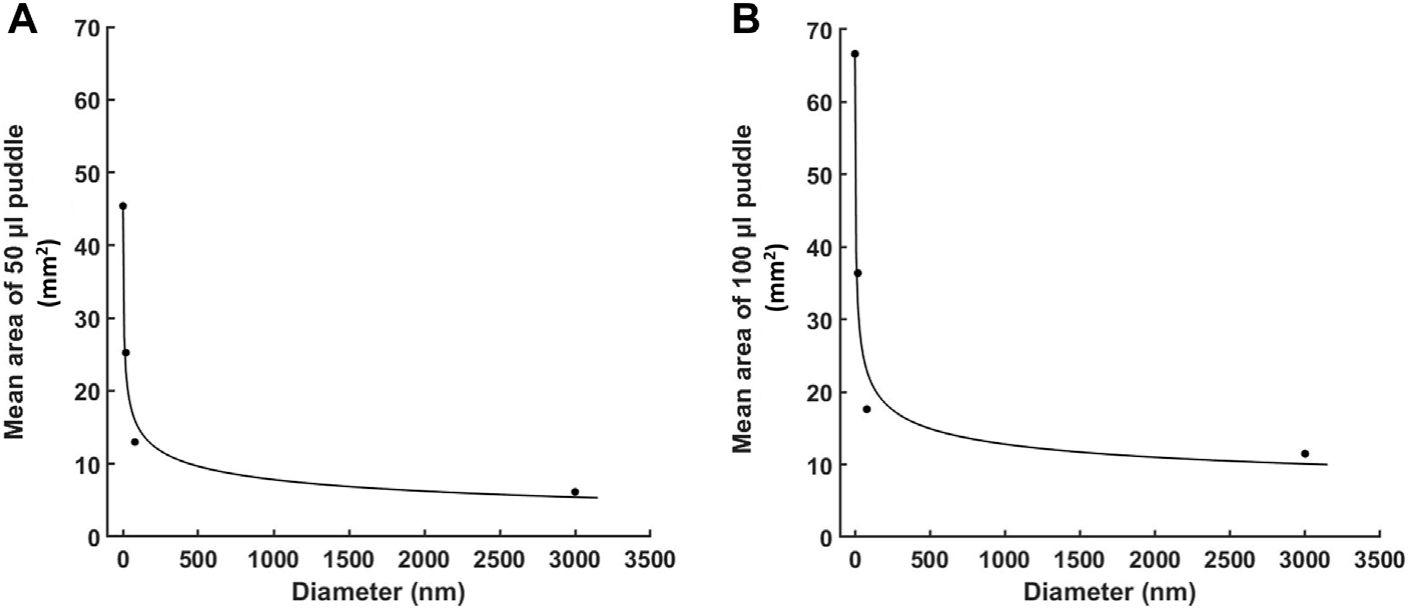
Quantitative curve fitting for the area of the puddle with respect to the particle’s diameter. A two-term power law fitting for the mean area of the puddle for: **(A)** 50 µL injections, and **(B)** 100 µL injections. Each dotted point represents the mean puddle area for one of the four molecules (n = 6 each). Fitting is generated by MATLAB.

Figure 5 shows a quantitative curve fitting model for the mean area of the puddle with respect to the particle’s diameter. Both 50 and 100 µL injections, for all materials, obey a two-term power law function: f (x) = a·x^b^ + C, with R-square values of .9817 and .973, respectively for the values of the coefficients as presented in Table 2. These values are a strong indication of the validity of the fitting model, which is also reflected in Figures 5A, B where the puddle mean values are aligned with the fitting curves.

**TABLE 2:** Values for the power law coefficients with R-square values presenting quality of fitting. Coefficients of the power law fitting, f (x) = a·x^b^ + C, with R-square values presenting quality of fitting.

Injection (µL)	a	b	C	R-square
50	49.64	−.2084	−3.934	.9817
100	65.34	−.2576	1.792	.973

**FIGURE 6:**
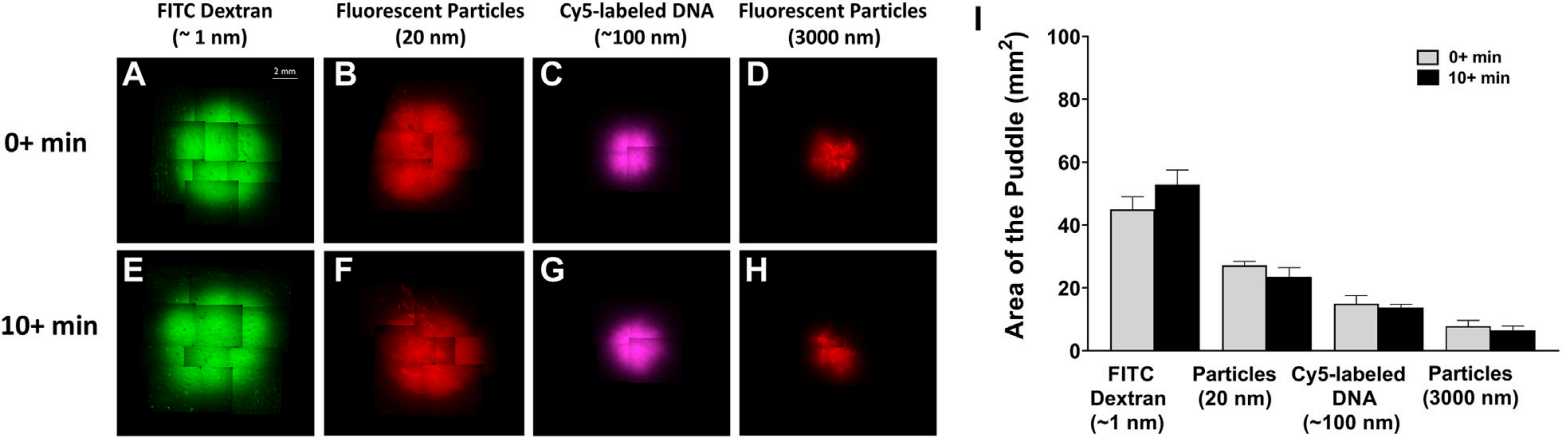
Puddle dispersion demonstrates no time dependence. ×4 objective top-view fluorescent images of the puddle momentarily 0 + min, **(A–D)** or 10 + min, **(E–H)** post 50 µL injection of the different material solutions. **(I)** Area quantification of the puddles, represented by mean ± SD. *n =* 3 each. No statistical significance is detected between areas of the different time points.

We also examined if cargo distribution pattern changes with respect to time. Figure 6 compares puddle size with 50 µL injection in two separate time frames: 1) 0 + refers to excising the skin sample momentarily after injection and 2) 10 + refers to excising the skin samples 10 min after injection. For all injection materials, no sign of further dispersion/diffusion was observed after a 10 min delay, and no statistically significant differences were detected for the same injection material.

## Discussion

With the increasing attention to and demand for dermal drug delivery therapeutics and approaches, identifying the location of the injected cargo puddle is critical to effectively and accurately target the drug injected into the skin (Kis et al., 2012; Gary and Weiner, 2020; Eusebio et al., 2021; Lallow et al., 2021; Tebas et al., 2021; Jeong et al., 2022; Lawal et al., 2022; Suschak et al., 2022). In this study, we attempt to characterize the location and size of the puddle in relation to the visible bleb formation after ID injection in a rat model. Utilizing four distinct size molecules (∼1 nm FITC-dextran, 20 nm fluorescent particles, ∼100 nm Cy5-labeled DNA, and 3 µm fluorescent particles), we have characterized: 1) two-dimensional area for the bleb and the puddle from a top view; 2) effect of injection volumes on bleb size; 3) puddle penetration depth; and 4) time-dependence for lateral dispersion of the puddle. These specific parameters are key factors for ID drug delivery, particularly for approaches that require a secondary delivery mechanism or when cellular uptake is required for function (Van Den Berg et al., 2009; Schultheis et al., 2018; Kim et al., 2019; Lallow et al., 2021; Momin et al., 2021; Benaouda et al., 2022; Jeong et al., 2022).

Several studies suggested that the injected payload directly colocalized with the visible injection bleb post ID injection (Chiu et al., 2007; Laurent et al., 2007; Kis et al., 2012), and as such concluded that the bleb site is a sufficient target for secondary delivery mechanisms. However, this work reveals that the molecule size of the cargo is a critical parameter in determining the size of the puddle, which may be different than the bleb size. Given the same injection volume, the size of the bleb is statistically uniform, regardless of the injected material. On the other hand, the size of the puddle decreases as the size of the molecule increases. This result is not surprising, as larger molecules tend to have less configurational mobility within the skin’s extracellular matrix (ECM) (Tomasetti and Breunig, 2018). In essence, the ECM acts as a steric filtration system entrapping larger molecules in a concentrated location while the liquid solvent creates the visible bleb area. In contrast, smaller molecules, as in the case of FITC-dextran, can disperse further through the skin exceeding the boundaries of the bleb, presumably *via* diffusion. This was evident for the 100 µL injection case where the area of the puddle was greater than that of the bleb indicating wider dispersion through the skin. In the vertical direction, a similar trend was observed where smaller molecules reached a greater depth compared to larger molecules. However, it is important to note that all injections appear to have penetrated to ∼2 mm within the skin. For typical applications, this depth sufficiently covers the densely cell-packed epidermal layer (∼200 µm) where uptake is the most active (White et al., 1999; Gurunathan et al., 2000; Wei et al., 2017). Lateral cargo distribution as observed from a 2D top-view is therefore the parameter most relevant to secondary deliveries. Of note, the fact that the vertical dispersion was minimally affected by the injection volume suggests that the epidermal and upper dermal cellular network forms a barrier against cargo movement in that direction; thus, to estimate lateral dispersion with varying injection volumes, one can essentially discount changes in cargo depth.

The areas of the puddle using different injection volumes (50 μL and 100 µL) are only statistically different for the smallest injected molecule, FITC-dextran; otherwise, they are considered the same. We did not consider greater injection volumes as the typical maximum volume for ID injection is around 100–200 µL (Criscuolo et al., 2019). Injection volumes greater than 100 µL frequently lead to fluid leakage out of the skin according to our experience. Our practice conforms with the Rutgers University IACUC Policy Handbook, which suggests the maximum ID injection for rats is 100 µL. Thus, for typical applications such as DNA delivery, injection volume has less effect on puddle size and distribution than the cargo’s molecular size. Regardless of the injection volume, however, the puddle size with respect to the molecular size follows very closely a power law distribution, which is clearly shown in Figures 5A, B. The ability to predict the area of the puddle for a specific molecular size is critical when determining the drug dose in patients and is convenient in situations where visualizing the area of the injected cargo distribution is difficult. For example, for melanoma treatments where intradermal injections are used at the location of the lesion (Sloot et al., 2016), larger chemotherapeutic molecules will have limited mobility within the lesion, thus leading to limited puddle size and as such may require multiple injections to ensure sufficient lateral dispersion spanning the entire lesion.

Specific to DNA-based vaccine delivery, establishing a baseline characterization for the puddle is essential to successfully targeting the injected drug and focusing secondary applications onto it, which is required for *in vivo* cellular transfection. Mechanisms utilizing microneedle arrays (MNs), such as electroporation, should take into consideration the effective array size, the spacing between the needles, and the length of the needles to direct the electric field (Van Den Berg et al., 2009; Gupta et al., 2020; Dong and Chang, 2021; Lawal et al., 2022). Other mechanisms, such as suction-mediated delivery, need an effective cup size area that colocalizes with the cargo location (Lallow et al., 2021; Jeong et al., 2022). Chemical approaches to enhancing the uptake of DNA-based vaccines do not require a secondary physical application, but rather utilize delivery cargos such as chitosan and polyethylenimine (PEI) (Kudsiova et al., 2008; Li et al., 2012). Such delivery cargos have a size range of 10–1,000 nm, which falls within the size range of the materials employed in this study (Khanmohammadi et al., 2015; Hu et al., 2021). Prediction of the lateral distribution of such delivery cargos should follow the power law distribution presented in Table 2. Regardless of the specific mechanism, the ability to target the puddle location rather than the visible bleb is critical for achieving high transfection efficiency and generating optimal drug delivery systems. The current puddle-based results help enable more precise approaches.

Another important parameter to consider in this context is the delay between ID injection and the application of the delivery mechanism. Drug delivery approaches, such as electroporation and suction-mediated delivery, apply their secondary delivery mechanism immediately post injection (Roos et al., 2009; Amante et al., 2015; Lallow et al., 2021; Jeong et al., 2022). Indeed, this has the obvious clinical advantage of limiting the waiting time between injection and treatment for the patient. Here, we have confirmed that a delay up to 10 min yielded no evidence of further cargo dispersion.

To summarize, we have demonstrated that for ID injections, the location of the injected cargo only partially overlaps with location of the visible bleb, and the location is rather dependent on the molecular size of the injected material and the injection volume. We have quantified puddle areas as a function of molecular/particle size, where the general trend is that puddle areas decrease and molecular/particle size increases. As the puddle size can be significantly smaller than bleb size, targeting the bleb for transfection of cells as using electroporation electrodes and or suction device can easily miss the puddle so that colocalization effects are not achieved. Colocalization is also an important consideration for biologic substances that directly target cells, such that the dispersion volume must be considered as a function of molecular size of the injected material. Similar trends are found in the direction vertical to the skin surface, but in all cases the puddle well-includes the epidermal layer where most active uptake occurs. Puddle dispersions reach a steady state momentarily post injection, which is not altered by further wait time. Optimization efforts for ID drug delivery systems with secondary delivery mechanisms such as DNA-based vaccines and therapeutics are highly encouraged to develop basic characterization focusing on the location of the puddle rather than that of the bleb. This can be extrapolated to other routes of injections (IM and SC), where the size and the location of the injected molecules are of functional necessity.

## Data Availability

The original contributions presented in the study are included in the article/Supplementary Material, further inquiries can be directed to the corresponding authors.
