# Supplementary material for: Molecular distribution in intradermal injection for transfer and delivery of therapeutics
Source: Front Drug Deliv. 2023 Jan 13;3:1095181. doi: 10.3389/fddev.2023.1095181 (PMC12363334; doi:10.3389/fddev.2023.1095181)
Supplement: Supplementary file 1 [file DataSheet1.pdf]

Supplementary Materials

Supplementary Materials for “Molecular Distribution in Intradermal Injection for Transfer and Delivery of Therapeutics”

Emran O. Lallow[1], Kishankumar J. Busha[2], Sarah H. Park[3], Maria Atzampou[2], Nandita C. Jhumur[1], Yasir Demiryurek[4], Christine C. Roberts[5], Jerry W. Shan[1], Jeffrey D. Zahn[2], David I. Shreiber[2], Young K. Park[5], Jonathan P. Singer[1], Joel N. Maslow[5*], and Hao Lin[1*]

*Email: hlin@soe.rutgers.edu

jmaslow@genels.us

[1]Department of Mechanical and Aerospace Engineering, Rutgers, The State University of New Jersey, Piscataway, NJ, United States.
[2]Department of Biomedical Engineering, Rutgers, The State University of New Jersey, Piscataway, NJ, United States.
[3]Department of Materials Science and Engineering, Rutgers, The State University of New Jersey, Piscataway, NJ, United States.
[4]Department of Mechanical Engineering, Temple University, Philadelphia, PA, United States.
[5]GeneOne Life Science Inc., Seoul, South Korea.

Exemplary results from the analysis algorithm developed in MATLAB

Figure S1 shows exemplary results from the analysis algorithm developed in MATLAB, in which the radial intensity for each case is inspected and an 80% intensity cutoff is consistently applied to each case.

Fig. S1. Exemplary results from the analysis algorithm developed in MATLAB.

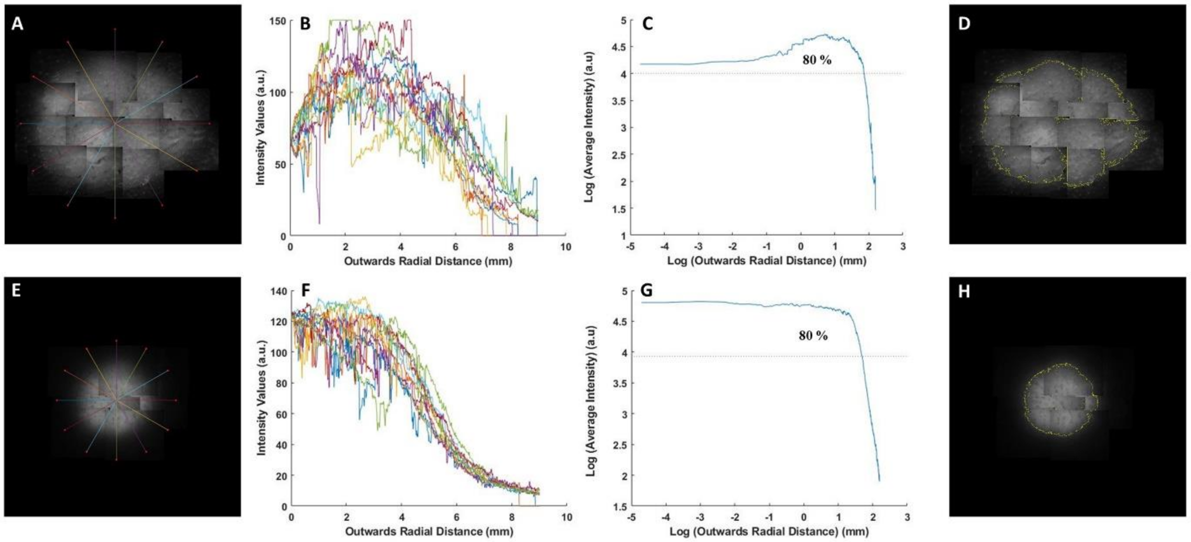

Figure S1: Exemplary results from the analysis algorithm developed in MATLAB. Exemplary results from the analysis algorithm for 100 µL injections of FITC-dextran (**A-D**) and Cy5-labeled DNA (**E-H**). **A** and **E** show 12 equidistant radial intensity lines going outwardly from the center of the puddle. **B** and **F** show the intensity values along each line. **C** and **G** show a (Log/Log) transformation of the average intensities of all lines with an 80% cutoff value determined by inspection. **D** and **H** show the captured puddle area after applying an 80% intensity cutoff.

DNA plasmids size measurements

Figure S2 represents DLS diameter measurements comparing Cy5-labeled DNA (99.83±14.45 nm) and unlabeled DNA (149.9±28.47) of the pEGFP-N1 plasmid.

Fig. S2. DNA plasmid size measurements.

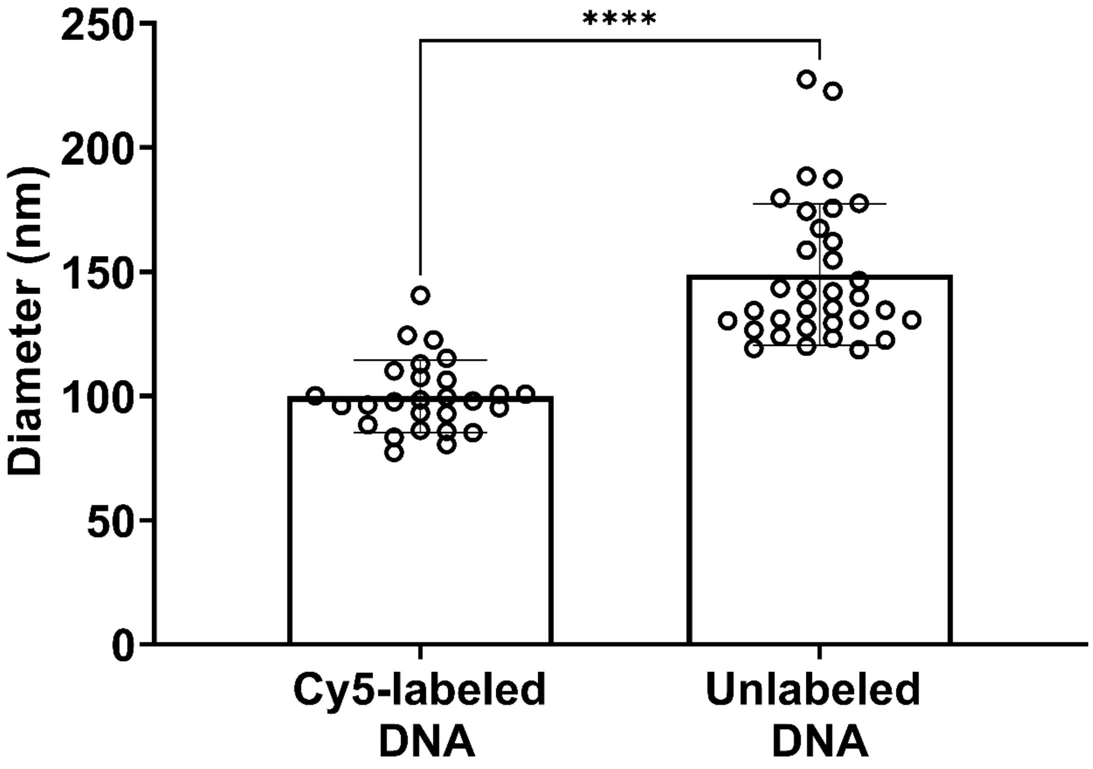

Figure S2: DNA plasmid size measurements. DLS diameter measurements for Cy5-labeled (n=27) and unlabeled DNA plasmids (n=34). Data represents mean ± SD. **** $p \leq 0.0001$ by student t-test.
